# Yalongjiang River Has Had an Important Role in the Dispersal and Divergence of *Rosa soulieana* in the Hengduan Mountains of China

**DOI:** 10.1371/journal.pone.0158586

**Published:** 2016-06-29

**Authors:** Hongying Jian, Yonghong Zhang, Xianqin Qiu, Huijun Yan, Qigang Wang, Hao Zhang, Hang Sun

**Affiliations:** 1 Key Laboratory of Biodiversity and Biogeography, Kunming Institute of Botany, Chinese Academy of Sciences, Kunming, China; 2 National Engineering Research Center for Ornamental Horticulture / Flower Research Institute, Yunnan Academy of Agricultural Sciences, Kunming, China; 3 Life Science Department, Yunnan Normal University, Kunming, China; University of Gottingen, GERMANY

## Abstract

The Hengduan Mountains are the core of the Sino-Himalayan Floristic Region. *Rosa soulieana* Crép. is an important wild rose species that is widely distributed in the Hengduan Mountains. To provide better future utilization of this wild rose, and also to add some possible proof of the effect of geomorphological and ecological characteristics of the Hengduan Mountains on the current spatial distribution and genetic diversity of local species, the genetic diversity and genetic structure of 556 individuals from 37 populations of *R*. *soulieana* were studied using fluorescent amplified fragment length polymorphisms (AFLPs). *R*. *soulieana* showed a moderately high level of genetic diversity and a high level of genetic differentiation at the species level. The total percentage of polymorphic loci, total heterozygosity (*H*t), Shannon index (*I*), and heterozygosity value within populations (*H*s) were 97.8%, 0.253, 0.339, and 0.139, respectively. More than half of the total genetic variation (54.0%) occurred within populations, and the overall gene differentiation coefficient (*G*st) was 0.451. The genetic differentiation among populations was positively and significantly correlated with geographic distance. The neighbor-joining cluster and the Bayesian analysis divided all the populations and individuals into 3 groups, and did not support the morphology based intraspecific varieties. The results confirmed that the ancient *R*. *soulieana* of the third group survived in northwestern Yunnan and Yalongjiang valley and then moved upnorth along the valley. The spatial distribution of the other two groups was the result of allopatric divergence due to long period of adaptation to the different climatic conditions of its distribution at either side of the Yalongjiang River.

## Introduction

Located at the south-eastern edge of the Qinghai-Tibetan Plateau in southwestern China, the Hengduan Mountains are characterized by a series of huge north-south ridges along rivers. The Hengduan Mountains area is a core region of the Sino-Himalayan Floristic Region [1], which is one of the world’s 25 biodiversity hotspots [2, 3]. The dramatic geomorphological and climatic changes from the Late Tertiary to the Quaternary, viz. the uplift of the eastern Qinghai-Tibetan Plateau since the Late Pliocene approximately 3.4 Mya ago and the climatic oscillations of the Pleiostocene (2.4–0.01 Mya) [4–8] in the Hengduan Mountains are assumed to be the most important factors influencing the current spatial distribution of local species and their genetic diversity [9]. There are 73 species/varieties and forms of wild roses mainly belonging to the section *Rosa* DC. (formerly *Cinnamomeae* DC.), the section *Synstylae* DC., the section *Pimpinellifoliae* DC. and the section *Microphyllae* Crép. in the Hengduan Mountains, 43 of which are endemic [10, 11]. However, very few studies have focused on the diversification, evolution and phylogeography of wild rose species in this biogeographically important area.

**Fig 1:**
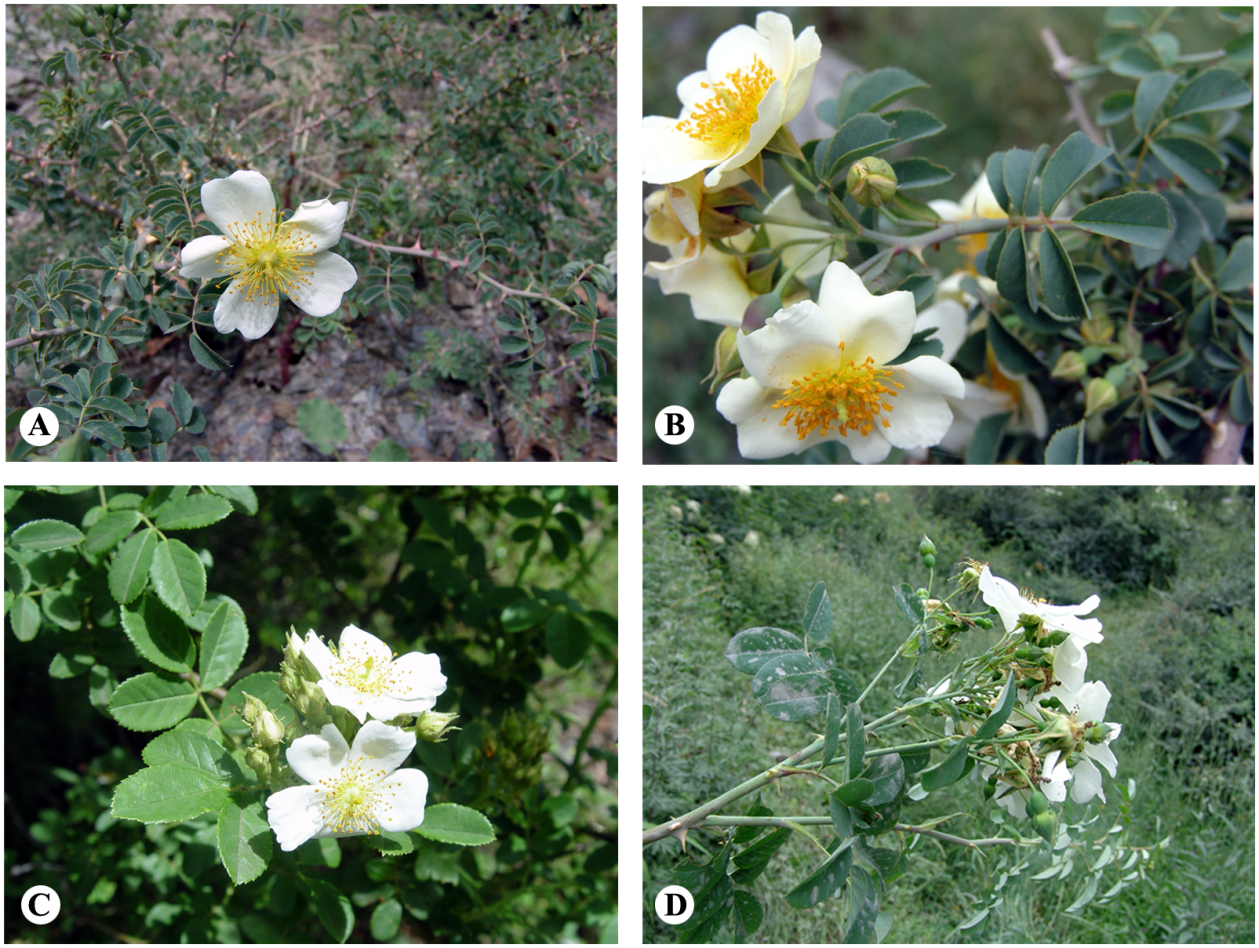
Four morphologically identified varieties of *Rosa soulieana*. (A) *R*. *soulieana* Crép.var. *microphylla* Yü et Ku. (B) *R*. *soulieana* var. *soulieana*. (C) *R*. *soulieana* var. *yunnanensis* Schneid. (D) *R*. *soulieana* var. *sungpanensis* Rehd.

*Rosa soulieana* Crép. is a hermaphroditic perennial shrub from the section *Synstylae* DC. in the genus *Rosa* L. [12]. It is widely distributed on the scrublands, slopes, stream sides, and farmlands along the dry and semi-dry valleys in the Himalaya–Hengduan Mountain (HHM) regions. It is conspicuous for its numerous white showy fragrant flowers in corymbs in the summer and for the large number of orange-red subglobose or ovoid shiny hips in autumn. *R*. *soulieana* is highly tolerant to the dry environment and can be potentially used for ecosystem restoration of the dry valleys [13]. In addition, its hips and pollen are rich in nutrients and phytocompounds [14]. Based on morphological variation in floret numbers in the corymb, pubescence of styles, pubescence and gland of rachis and abaxial sides of leaflets, gland of pedicels, and the size of leaflets, 4 varieties were recognized in this species (*R*. *soulieana* var. *microphylla* Yü et Ku, *R*. *soulieana* var. *sungpanensis* Rehd., *R*. *soulieana* var. *soulieana*, and *R*. *soulieana* var. *yunnanensis* Schneid.) (Fig 1) [12, 15]. The variety with larger leaves, *R*. *soulieana* var. *sungpanensis*, is limited to Songpan County of Northern Sichuan, and the variety with the smallest leaflets, *R*. *soulieana* var. *microphylla*, is limited to Baxoi County in Tibet [15]. The ranges of the other two varieties overlap in northwestern Yunnan, southeastern Tibet and western Sichuan. However, this morphology-based *R*. *soulieana* intraspecific classification was not supported by the cpDNA-based phylogeny [16], which can be due either to high morphological plasticity within the species or because of low resolution of the used molecular markers. Thus, genetically more variable markers are needed for robust conclusions.

Genetic variation at the intraspecific level is critical for the adaptation to environmental changes and for long-term survival of a species [17]. Knowledge of extent and structure of genetic diversity, and its causes can provide insight on species ecological and evolutionary histories [18, 19]. In addition, genetic variation of a species has profound implications for conservation and future breeding programs [20]. The nuclear genome has a larger effective population size and higher rate of dispersal compared to plastid genomes [17]. As a nuclear multi-locus survey technique, AFLP allows high-resolution genetic analysis of closely related individuals and populations [21]. Despite two weaknesses, a dominant mode of inheritance and the occurrence of size homoplasy [22], it has been successfully used for the analysis of genetic diversity and the population structure of many species [22–26].

As a fragrant wild rose species, which is also highly tolerant to the dry and barren environment, *R*. *soulieana* can be directly used both for the ecosystem revegetation of dry valleys and for landscape utilization. It can also be potentially used for improving modern rose cultivars in rose breeding programs. To provide better future utilization of this wild rose, and also to add some possible proof for the effect of the geomorphological and ecological characteristics of the Hengduan Mountains on the current spatial distribution and genetic diversity of local species, we use AFLPs analysis to study the genetic variation of *R*. *soulieana*, the aims of which are as the follows: 1) To determine the extent and structure of the genetic diversity of *R*. *soulieana* in the Hengduan Mountains Region. 2) To determine whether the intraspecific varieties are independent evolutionary units or just arbitrary units plastically responded to the geological and ecological environment in the Hengduan Mountains. 3) To assess possible causes of the existing genetic diversity and genetic structure of *R*. *soulieana*.

## Materials and Methods

### Plant materials

**Fig 2:**
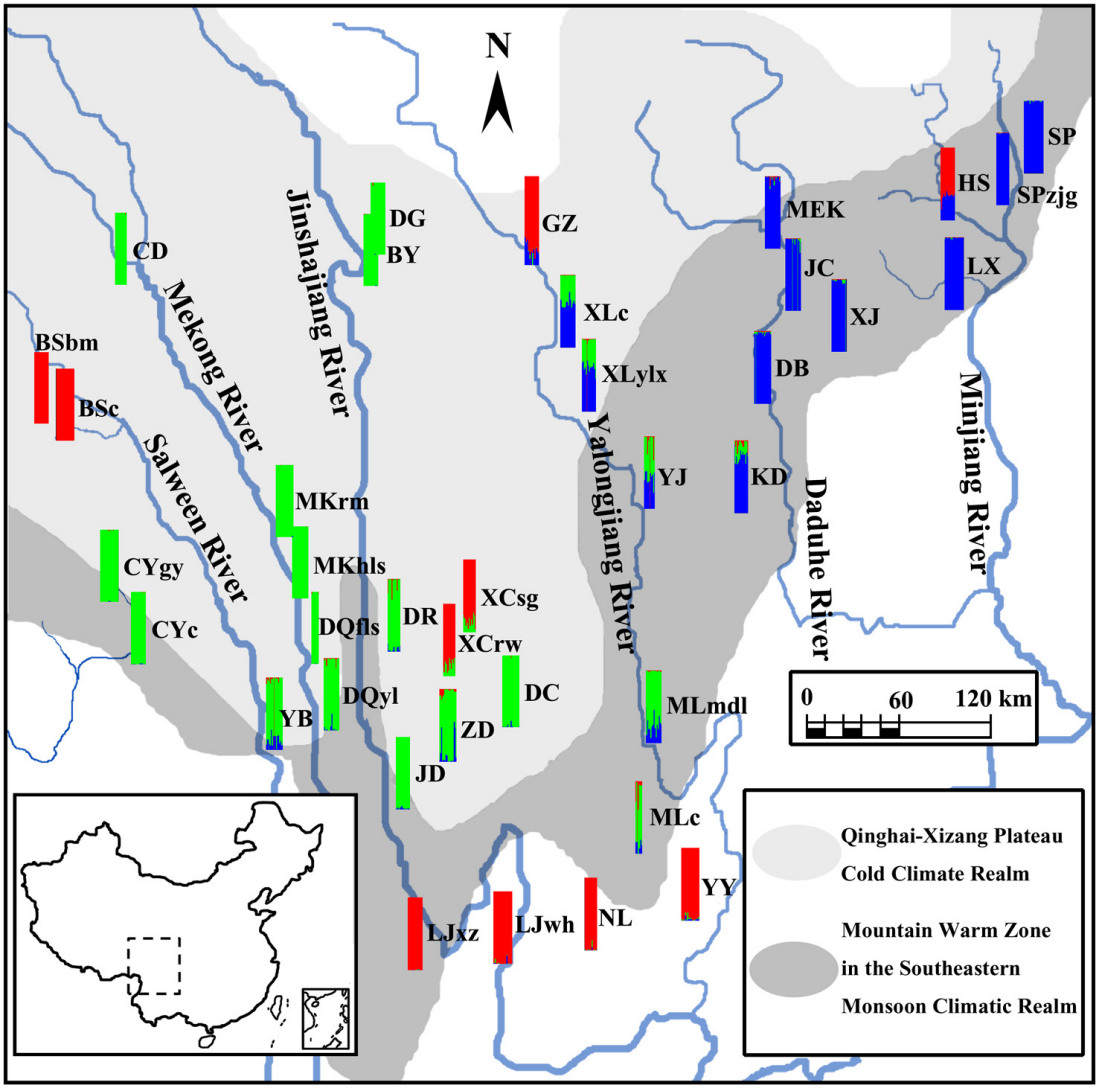
Distribution of the collected populations and spatial genetic structure of *Rosa soulieana*. Colors indicate genetic partitions for individuals as revealed by Bayesian analysis.

In total, 556 individuals representing 37 populations were sampled, covering the known distribution area of *R*. *soulieana* in the Hengduan Mountains and Zayü in the eastern Himalayas, southwestern China (Table 1, Fig 2). It is not an endangered or protected plant and the field collection was permitted by the local Forestry Administration. Clean and healthy young leaves were collected and dried in silica-gel during the field expeditions from June 15 to July 30 in 2010. The randomly sampled individuals were at least 20 m apart from each other. The latitude, longitude and altitude of each population were recorded using an eTrex Global Positioning System (Garmin, Taiwan). The specimens were deposited in the herbarium of KUN.

**Table 1:** Details of sample locations, sample sizes, number of polymorphic loci (*NP*), percentage of polymorphic loci (*P*), Nei’s gene diversity (*He*) and Shannon’s information index (*I*) in37 populations of *Rosa soulieana*.

Pop.	Variety	Locality	Voucher specimen	Latitude	Longitude	Altitude	Sample	*NP*	*P* %	*He* (St.Dev.)	*I* (St.Dev.)
BSbm	*microphylla*	Baxoi 1, Tibet	Jm087, KUN	30°00′56.4″	97°03′47.4″	3056	15	126	30.2	0.104(0.174)	0.156(0.253)
BSc	*soulieana*	Baxoi 2, Tibet	Jm104, KUN	30°03′51.1″	96°56′12.0″	3221	18	140	33.6	0.110(0.179)	0.165(0.258)
BY	*soulieana*	Baiyü, Sichuan	Jm107, KUN	31°22′53.2″	98°53′20.7″	2987	15	120	28.8	0.099(0.174)	0.147(0.252)
CD	*soulieana*	Qamdo, Tibet	Jm105, KUN	31°10′53.7″	97°05′47.1″	3279	12	133	31.9	0.112(0.183)	0.167(0.264)
CYc	*yunnanensis*	Zayü 1, Tibet	Jm095, KUN	28°37′12.0″	97°29′24.0″	2300	15	173	41.5	0.374(0.189)	0.207(0.273)
CYgy	*soulieana*	Zayü 2, Tibet	Jm096, KUN	28°55′39.0″	97°25′49.6″	2714	18	177	42.5	0.132(0.183)	0.200(0.265)
DB	*soulieana*	Danba, Sichuan	Jm139, KUN	30°49′24.4″	101°56′15.4″	1839	17	154	36.9	0.116(0.177)	0.177(0.257)
DC	*soulieana*	Daocheng, Sichuan	Jm067, KUN	28°54′00.4″	100°16′54.9″	3000	18	190	45.6	0.148(0.190)	0.223(0.274)
DG	*soulieana*	Deger, Sichuan	Jm106, KUN	31°37′38.4″	98°35′22.5″	3043	15	131	31.4	0.104(0.176)	0.156(0.254)
DQfls	*yunnanensis*	Deqin 1, Yunnan	Jm082, KUN	28°29′17.7″	98°50′01.2″	2883	8	179	42.9	0.148(0.194)	0.223(0.278)
DQyl	*soulieana*	Deqin 2, Yunnan	Jm078, KUN	28°19′02.7″	98°53′59.0″	2645	16	232	55.6	0.180(0.196)	0.272(0.280)
DR	*soulieana*	Derong, Sichuan	Jm075, KUN	29°04′17.7″	99°22′55.7″	3272	13	245	58.8	0.202(0.201)	0.303(0.287)
GZ	*soulieana*	Ganzi, Sichuan	Jm118, KUN	31°27′39.2″	100°07′01.9″	3298	12	174	41.7	0.140(0.192)	0.210(0.275)
HS	*soulieana*	Heishui, Sichuan	Jm158, KUN	31°56′46.9″	103°24′53.7″	1797	15	154	36.9	0.110(0.172)	0.168(0.250)
JC	*soulieana*	Jinchuan, Sichuan	Jm143, KUN	31°21′29.9″	102°59′24.9″	2130	16	146	35.0	0.111(0.177)	0.169(0.256)
JD	*yunnanensis*	Shangrila 1, Yunnan	JQ032, KUN	27°57′4.78″	99°24′59.5″	1900	15	214	51.3	0.167(0.195)	0.252(0.279)
KD	*soulieana*	Kangting, Sihcuan	Jm132, KUN	30°00′37.8″	101°57′06.6″	2803	14	201	48.2	0.156(0.193)	0.236(0.277)
LJwh	*yunnanensis*	Lijiang 1, Yunnan	Jm034, KUN	26°57′12.5″	100°11′17.4″	2800	19	283	67.9	0.216(0.196)	0.326(0.277)
LJxz	*yunnanensis*	Lijiang, Yunnan	Jm040, KUN	27°12′20.4″	99°26′38.4″	2500	15	157	37.7	0.106(0.170)	0.164(0.246)
LX	*soulieana*	Lixian, Sichuan	Jm154, KUN	31°28′49.6″	103°11′22.1″	1790	19	137	32.9	0.108(0.176)	0.163(0.256)
MEK	*soulieana*	Barkam, Sichuan	Jm144, KUN	31°55′46.3″	102°06′55.6″	2512	16	167	40.1	0.130(0.185)	0.197(0.268)
MKhls	*soulieana*	Markam1, Tibet	Jm083, KUN	29°11′08.8″	98°37′52.6″	3340	17	197	47.2	0.155(0.194)	0.233(0.278)
MKrm	*soulieana*	Markam2, Tibet	Jm085, KUN	29°42′00.9″	98°24′15.8″	3304	18	199	47.7	0.154(0.188)	0.234(0.273)
MLc	*yunnanensis*	Muli 1, Sichuan	Jm015, KUN	27°56′07.3″	101°17′25.7″	2357	6	166	39.8	0.139(0.191)	0.209(0.275)
MLmdl	*soulieana*	Muli 2, Sichuan	Jm014, KUN	28°35′37.2″	101°13′19.3″	2006	16	193	46.3	0.162(0.198)	0.242(0.285)
NL	*yunnanensis*	Ninglang, Sichuan	Jm031, KUN	27°28′39.4″	100°46′16.8″	2300	13	176	42.2	0.144(0.195)	0.216(0.279)
SP	*soulieana*	Songpan 2, Sichuan	Jm162, KUN	32°22′38.0″	103°43′50.6″	2530	20	143	34.3	0.114(0.181)	0.171(0.262)
SPzjg	*sungpanensis*	Songpan 1, Sichuan	Jm163, KUN	32°22′38.0″	103°43′50.6″	2530	11	101	24.2	0.085(0.167)	0.127(0.241)
XCrw	*soulieana*	Xiangcheng1, Sichuan	Jm059, KUN	28°44′40.8″	99°50′53.5″	3550	13	188	45.1	0.150(0.193)	0.226(0.278)
XCsg	*soulieana*	Xiangcheng2, Sichuan	Jm069, KUN	29°07′42.7″	99°54′45.7″	2700	13	186	44.6	0.156(0.196)	0.233(0.283)
XJ	*soulieana*	Xiaojin, Sichuan	Jm140, KUN	31°06′30.5″	102°25′51.7″	2465	15	161	38.6	0.116(0.179)	0.177(0.257)
XLc	*soulieana*	Xinlong1, Sichuan	Jm120, KUN	30°57′40.2″	100°19′23.9″	3050	16	161	38.6	0.132(0.191)	0.196(0.274)
XLylx	*soulieana*	Xinlong2, Sichuan	Jm124, KUN	30°31′11.8″	100°25′30.1″	3387	14	179	42.9	0.144(0.192)	0.216(0.276)
YB	*yunnanensis*	YuBeng, Yunnan	Jl020, KUN	28°23′27″	98°47′34″	2950	17	186	44.6	0.144(0.191)	0.218(0.275)
YJ	*soulieana*	Yajiang, Sichuan	Jm128, KUN	30°04′34.3″	101°06′26.4″	2817	11	222	53.2	0.194(0.207)	0.286(0.295)
YY	*yunnanensis*	Yanyuan, Sichuan	Jm011, KUN	27°30′51.4″	101°41′29.7″	2700	18	157	37.7	0.120(0.180)	0.181(0.261)
ZD	*soulieana*	Shangrila, Yunnan	Jm053, KUN	28°01′13.9″	99°43′2.3″	2950	17	222	53.2	0.185(0.223)	0.277(0.289)
Mean								173.6	41.5	0.145(0.170)	0.209(0.234)
Total							557	408	97.8	0.253(0.029)	0339(0.011)

### DNA extraction and AFLP genotyping

The total genomic DNA of the collected specimens was extracted with Plant DNA-Easy kit (Bioteke Cooperation, Beijing, China). AFLP reactions were performed according to Vos et al.[21] with some modification. The modification was that the *EcoR* I primers for selective amplification were labeled fluorescently and the final PCR products were analyzed on an ABI 3100 automated DNA sequencer (Applied Biosystems, CA, USA). The detailed procedures were as the follows. For the digestion, a 30 μl volume of digestion mix containing 4 μl NEBuffer (New England Biolabs, MA, USA), 3 U *EcoR* I, 1.5 U *Mse* I, and 4 μl 10 mg/ml BSA was added into 10 μl of genomic DNA (approximately 100 ng). The reaction was incubated at 37°C for 1.5 h. Next, 10 μl ligation mix, which included 3 μl 5μM *EcoR* I adaptor, 3 μl 5 μM *Mse* I adaptor, 1 μl 10×Ligase Buffer and 5 U T4 ligase, was incubated at 65°C for 10 min and then slowly reduced to room temperature in the machine. Then, the ligation mix was added to the 40 μl restriction digested DNA and kept at 16°C for 3 h. After ligation, the mix was diluted 10-fold with sterile deionized water (sdH_2_O). A pre-selective polymerase chain reaction (PCR) was done using a Bio-Rad machine with a single selective nucleotide extension. The reaction mix (total volume of 20 μl) included 5 μl diluted restriction/ligation temple DNA, 0.5 U Ex-*Taq* polymerase, 2 μl 10×PCR buffer (Takara) (Mg^2+^ free), along with 1.2 μl MgCl_2_, 1 μl 10 μM *EcoR* I Primer (+1), 1 μl 10 μM *Mse* I Primer (+1), and 1.6 μl 2.5 mM dNTPs (Takara). After an initial incubation at 65°C for 5 min, 30 cycles of 94°C for 30 s, 56°C for 30 s and 72°C for 1 min were performed with a final extension for 5 min. We electrophoresed 5 μl pre-amplified product on a 1.0% agarose gel to identify the most variable selective primer extensions. The pre-amplified products were then diluted 10-fold with sdH_2_O and stored at -20°C. Four selective primer pairs: *EcoR* I (FAM) -AAG/ *Mse* I -CAT, *EcoR* I (FAM) -AAG/*Mse* I- CGT, *EcoR* I (FAM) -AAG/*Mse* I- CTC and *EcoR* I (FAM)-AAG/*Mse* I–CTT (FAM is a kind of fluorescent dye; primer sequences are listed in Table 2) were selected for the amplification reaction. The reaction mix (total volume of 20 μl) included 5 μl 10-fold diluted pre-PCR product, 0.5 U Ex-*Taq* polymerase, 2 μl 10×PCR buffer (Takara) (Mg^2+^ free), along with 1.2 μl MgCl_2_, 1 μl 10 μM *Eco*R I Primer (+3), 1 μl 10 μM *Mse* I Primer (+3), and 1.6 μl 2.5 mM dNTPs (Takara). After an initial incubation at 94°C for 2 min, 13 cycles of 94°C for 30 s, 65°C and touch down 0.7°C for each cycle (the annealing temperature was successively reduced by 0.7°C.) for 30 s, and 72°C for 1min, 23 cycles of 94°C for 30 s, 56°C for 30 s and 72°C for 1min were performed with a final extension for 5 min. Then, 5 μl final selectively amplified product was electrophoresed on a 1.0% agarose gel to determine if the length of the amplified product was approximately 100 bp-500 bp.

**Table 2:** Number of loci (N), number of polymorphic loci (*NP*), and the percentage of polymorphic loci (*P*) detected by each selective primer combination.

Code	Selective primer combination	N	*NP*	*P* (%)
1	*EcoR* I–AAG/*Mse* I–CAT	102	99	97.1
2	*EcoR* I–AAG/*Mse* I–CGT	110	105	95.5
3	*EcoR* I–AAG/*Mse* I–CTC	96	95	99.0
4	*EcoR* I–AAG/*Mse* I–CTT	109	109	100.0
Total		417	408	97.8

The amplicons were detected using an ABI 3100 capillary sequencer (Applied Biosystems) by MicroRead Gene Technique Company in Beijing. For each individual, 2 μl of the AFLP products were separately combined with 0.5 μL GeneScan ROX 500 (Applied Biosystems) as an internal size standard (Applied Biosystems) and run on the sequencer. In order to estimate the stability of band pattern, 5 samples in each plate were repeated during selective amplification and capillary sequencing. Electropherograms were analyzed using GeneMarker software v2.2.0 (SoftGenetics LLC, PA, USA) using the default parameters recommended as optimal for AFLP markers by the manufacturer (http://www.softgenetics.com/GeneMarker). Peak patterns were converted to dominant presence—absence (1–0) matrices. Electropherograms were checked manually to exclude doubtful peaks, and only peaks with sizes between 100 bp-500 bp were included in the analysis.

### Data analysis

Such indices of genetic diversity as the number of polymorphic loci (*NP*), the percentage of polymorphic loci (*P*), Nei’s [27] unbiased expected heterozygosity (*H*e) assuming Hardy–Weinberg equilibrium, and Shannon’s diversity (*I*) [28] were calculated for each population with POPGENE Version 1.31 [29]. At the species level, we calculated the percentage of polymorphic loci (P), total heterozygosity (*H*t), heterozygosity within populations (*H*s), and gene differentiation coefficient (*G*st) using the same software.

To assess the hierarchical genetic structure among populations and within populations, the AMOVA analysis was first performed by partitioning genetic variation among and within populations at the species level using ARLEQUIN version 3.0 [30]. The amount of gene flow among populations was estimated as *N*m = (1/*G*_st_- 1)/4 [31].

A data matrix of pairwise *F*_st_ values between populations and bootstrap values (1,000 permutations) were calculated using the program AFLP-SURV version 1.0 [32]. These distance matrices were then used as input files for the PHYLIP 3.6 software [33]. The neighbor-joining (NJ) trees of populations were produced with the NEIGHBOR program and visualized in TreeView (version 1.6.6) [34]. The populations were assigned to the groups identified by the cluster analysis (NJ tree). ARLEQUIN version 3.0 [30] was also used to determine if there was genetic differentiation among the groups. The percentage of polymorphic loci (P), total heterozygosity (*H*t), and heterozygosity within populations (*H*s) were further calculated for each group using POPGENE Version 1.31[29].

To assess the correlation between geographic and Nei’s genetic distances, the Mantel [35] test was performed with the software GENEALEX version 6.0 [36]. This software was also used to perform a Principal Coordinate analysis (PCoA) to assess the genetic similarity of the individuals and populations, respectively.

Finally, we examined the assignment of individuals to groups based on their multilocus genotypes using the Markov Chain Monte Carlo (MCMC) Bayesian clustering method in STRUCTURE version 2.2 [37]. The ‘admixture’ model was used and ‘Allele Frequencies are Correlated among Populations’ was assumed for the analysis. The model was run for 10,000 iterations after a burn-in period of 10,000. Analyses for the predefined value of k (number of groups) were run 10 times for 2≤k≤15 to ensure consistent results. The best estimation of k for the data set is usually selected by choosing the model that gives both the highest probability of the data and consistent results after multiple runs. The most probable k value was decided according to the rate of change in probability (ΔK) between successive K values, as proposed by Evanno et al. [38].

## Results

### DNA marker profile and population genetic diversity

The four AFLP primer combinations produced a total of 417 fragments in 556 individuals from 37 populations, 408 of which were polymorphic (Table 2, S1–S4 Tables, doi:10.5061/dryad.4q53p). The number of scored bands for each primer combination varied from 96 to 109 with a mean of 104.3 bands per primer. The maximum number of polymorphic bands was produced by primer combination *EcoR* I-AAG/*Mse* I–CTT (109 bands). The percentage of polymorphisms was from 95.5% to 100% with an average polymorphism percentage of 97.8%; the highest percentage of polymorphism was also produced by primer combination *EcoR* I-AAG/*Mse* I–CTT (100%).

As shown in Table 1, the number of polymorphic loci (*NP*) per population varied from 101 (SPzjg) to 283 (LJwh), with an average of 173.6 loci per population. The percentage of polymorphic bands (*P*) ranged from 24.2% (SPzjg) to 67.9% (LJwh), with an average of 41.5%. At the population level, the Nei’s gene diversity (*He*) of population varied from 0.085 (SPzjg) to 0.374 (CYc), with an average of 0.145. Population SPzjg, BY, BSbm, DG, LJxz, LX, HS and BSc had the lowest gene diversity, less than 0.110, while population JD, DQyl, ZD, YJ, DR, LJwh and CYc had the highest gene diversity, greater than 0.160. As far as Shannon’s information index (*I*) was concerned, it ranged from 0.127 (SPzjg) to 0.329 (LJwh), with an average of 0.209. Similar to Nei’s gene diversity, population SPzjg, BY, BSbm, DG, LX, LJxz, and BSc had the lowest Shannon’s information index, less than 0.165, while population MLmdl, JD, DQyl, ZD, YJ, DR and LJwh had the highest Shannon’s information index, greater than 0.242.

### Principal Coordinate analysis

**Fig 3:**
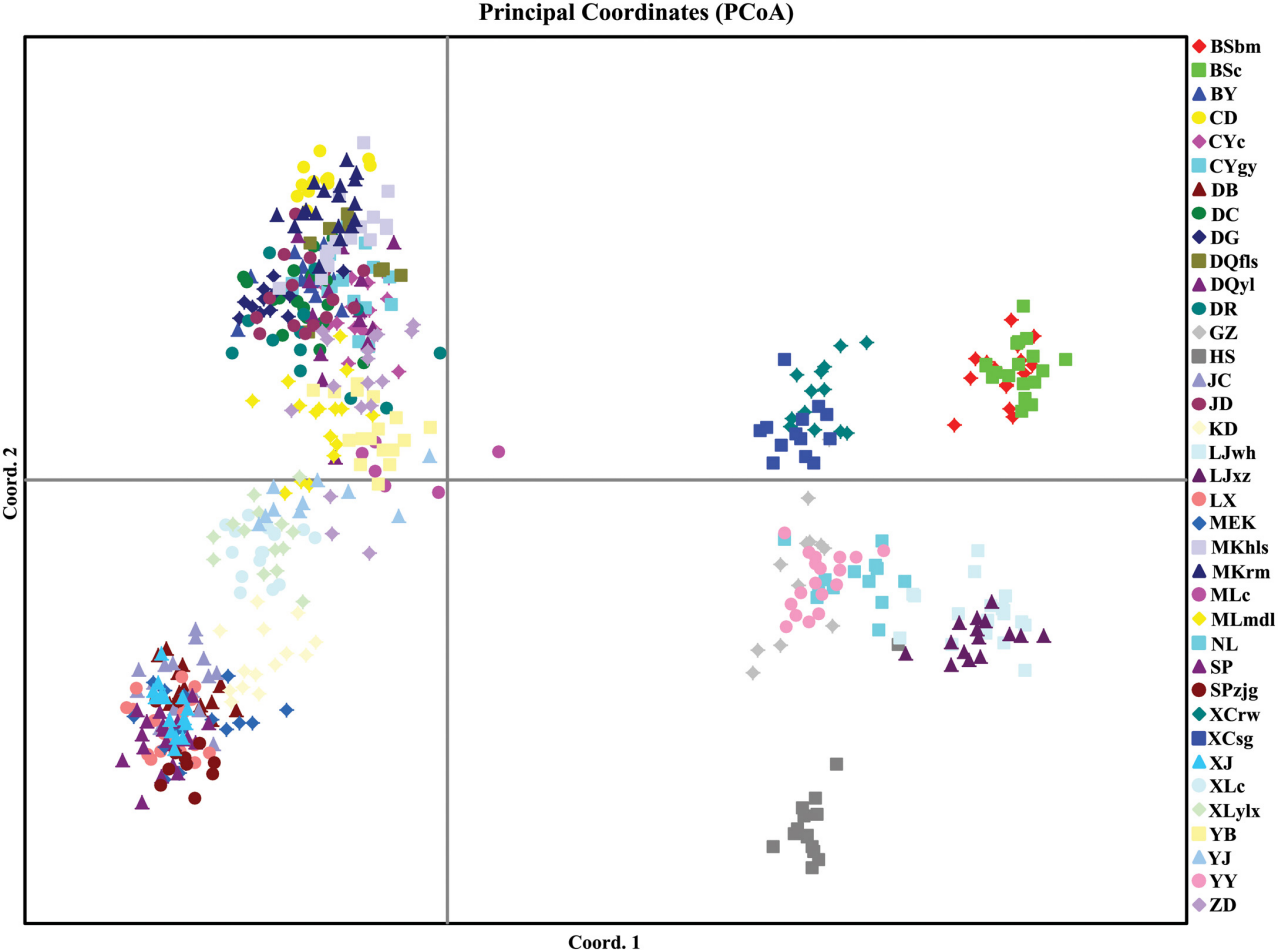
Principal Coordinate analysis of *R*. *soulieana* individuals. The first and second axis explained 13.0% and 7.8% of the total genetic similarities, respectively.

The first and the second axes of the Principal Coordinate analysis of 556 individuals from 37 populations explained 13.0% and 7.8% of the genetic similarities among all the individuals, respectively. As shown in Fig 3, in general, individuals originating in the same population were clustered together, with the exception that only a very few individuals from GZ, DR, ZD etc. were farther away from other individuals of the same population and clustered to other populations.

**Fig 4:**
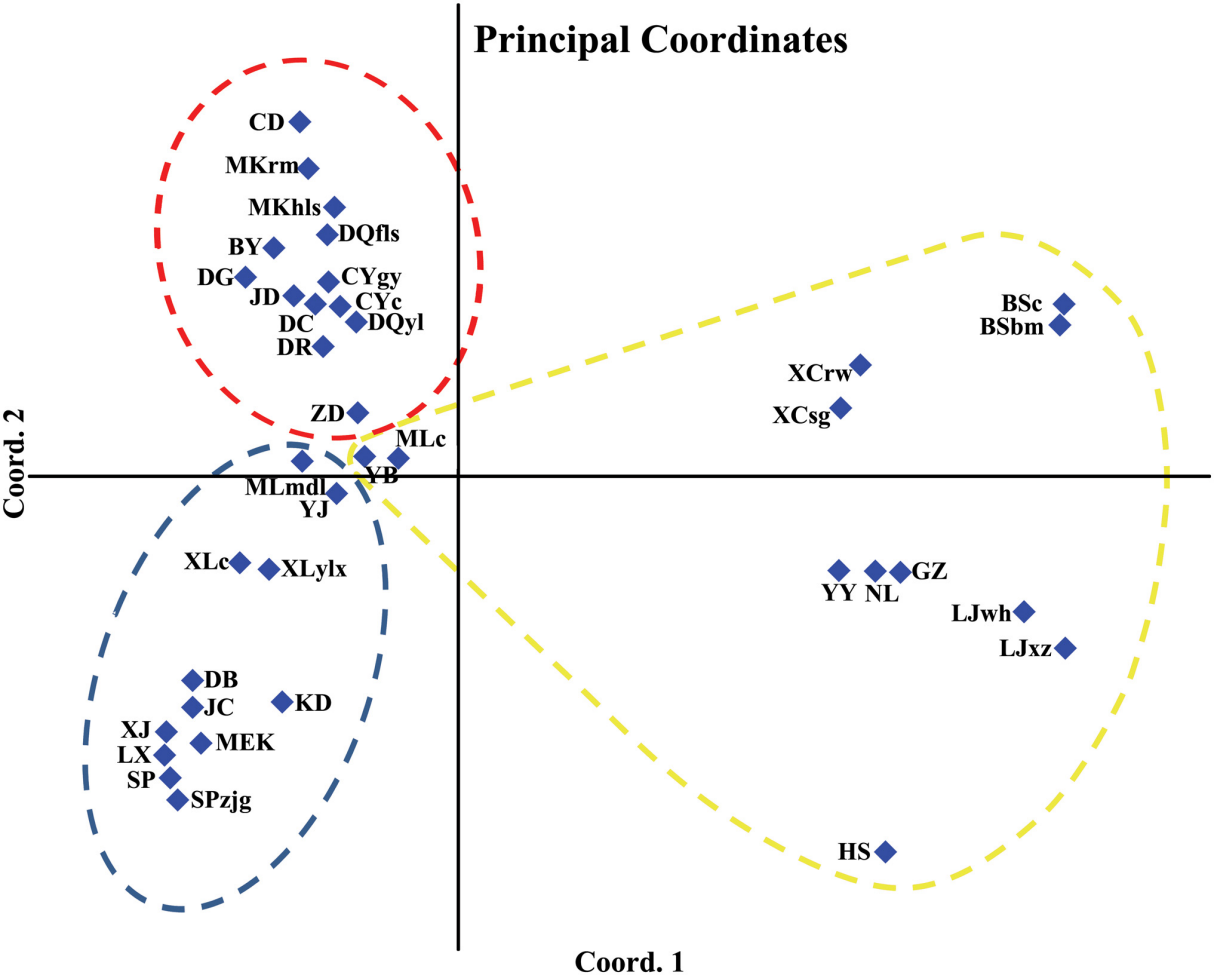
Principal Coordinate analysis of the *R*. *soulieana* populations. The first and the second axis explained 27.6% and 15.5% of the total genetic similarities, respectively. The blue, red and yellow dashed circles denote population groups I, II and III corresponding to the NJ clusters, respectively.

For the PCoA analysis of the 37 populations (Fig 4), the first and the second axis explained 27.6% and 15.5% of the similarities among all the populations, respectively. HS was far away from the other populations. BSbm and BSc, XCrw and XCsg, LJwh and LJxz, clustered to each other respectively. NL and YY were geologically close to each other and clustered together with GZ in the PCoA results. These scattered smaller groups composed a larger group (Fig 4), in which all the populations were at the edge of the species range except for XCrw and XCsg (Fig 2). Then, populations along the Mekong River including CD, MKrm, MKhls, DQfls, DQyl, populations along the Jinshajiang River including BY, DG, DR, JD, DC, and population s from the eastern Himalayan region including CYgy and CYc, clustered into another larger group (Figs 2 and 4). Meanwhile, populations along the Daduhe River, such as DB, JC, XJ, MEK, KD, and those along the Minjiang River, such as LX, SP, SPzjg, were clustered together. Others, including populations along the Yalongjiang River, e.g., MLmdl, MLc, YJ, XLylx and XLc, and population ZD close to Jinshajiang River and YB close to Mekong River, clustered together, among which XLylx and XLc were closer to populations of the Daduhe River, while the other 5 populations were closer to those from the Mekong and Jinshajiang River (Figs 2 and 4).

### Neighbor-joining tree construction and Bayesian analysis

**Fig 5:**
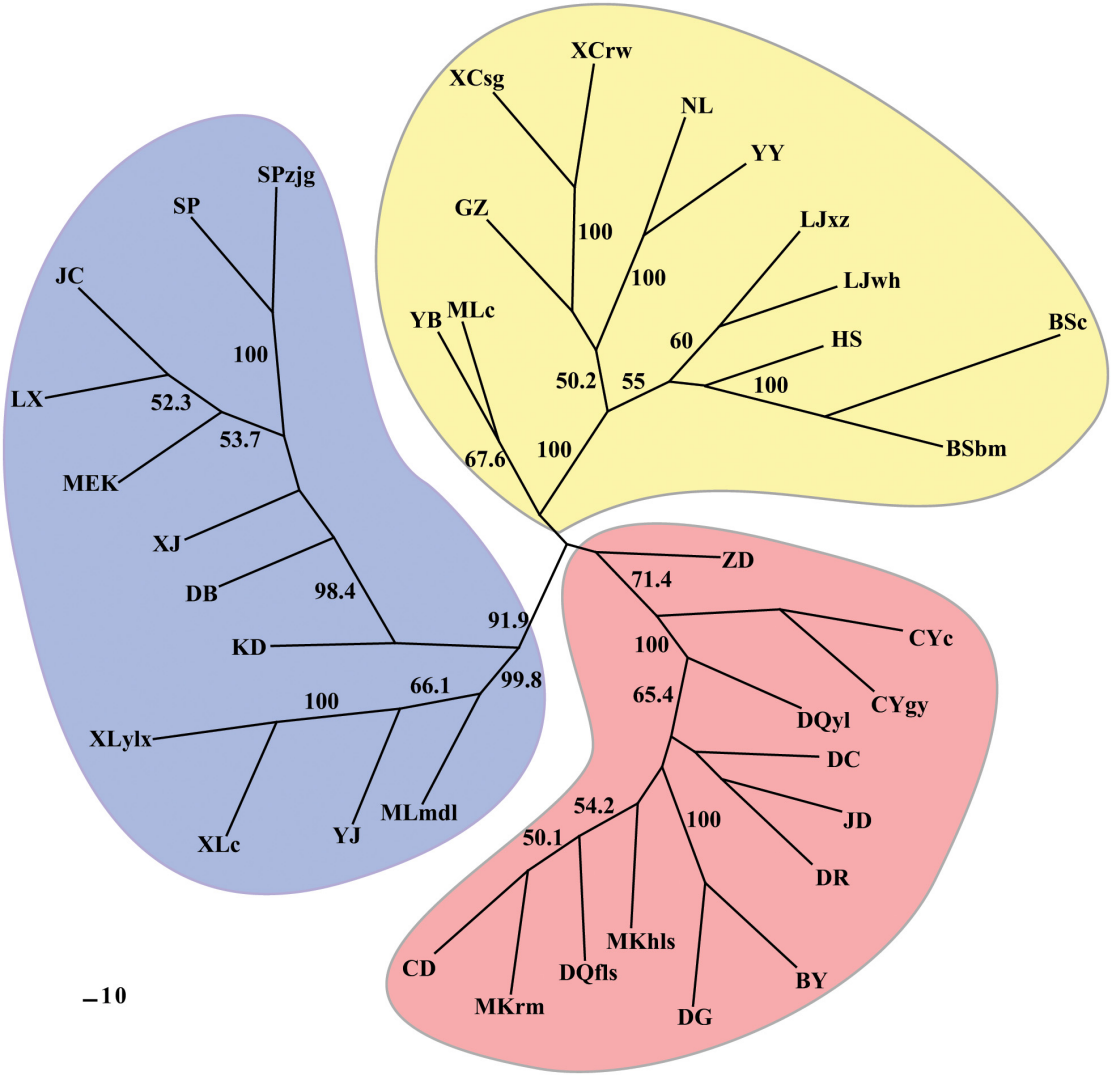
Neighbor joining tree of *R*. *soulieana* populations based on pairwise *F*_st_. The figures show the bootstrap values. The blue circle indicates group I, the pink circle indicates group II, and the yellow circle indicates group III.

Pairwise genetic distances between populations (*F*_st_) were calculated (S5 Table, doi:10.5061/dryad.4q53p). The neighbor-joining phylogram constructed from the population pairwise *F*_st_ value produced 3 groups (Fig 5). Group I included populations from Minjiang River drainage, i.e., SPzjg, SP, LX, populations from the Daduhe River drainage, i.e., MEK, JC, XJ, DB, and KD, populations from the Yalongjiang River drainage, i.e., XLc, XLylx, YJ and MLmdl. Group II, with a bootstrap lower than 50, included the population of CYc and CYgy from the eastern Himalayas, populations from the upper Mekong River, i. e., DQyl, DQfls, MKhls, MKrm and CD, and populations from the Jinshajiang River, i. e., DG, BY, DR, JD and DC. Group III comprised populations from the edge of the species range and populations from the lower part of Yalongjiang River, also with a bootstrap value lower than 50. In this group, HS resided the riverside of a branch of the Minjiang River in the northeastern of the species range. BSbm and BSc were at the northwestern of the range. GZ was at the uppermost of the Yalongjiang River. YB, XCrw, XCsg, ZD, MLc, LJxz, LJwh, YY, NL were at the southwestern of the species range and close to each other geologically.

**Fig 6:**
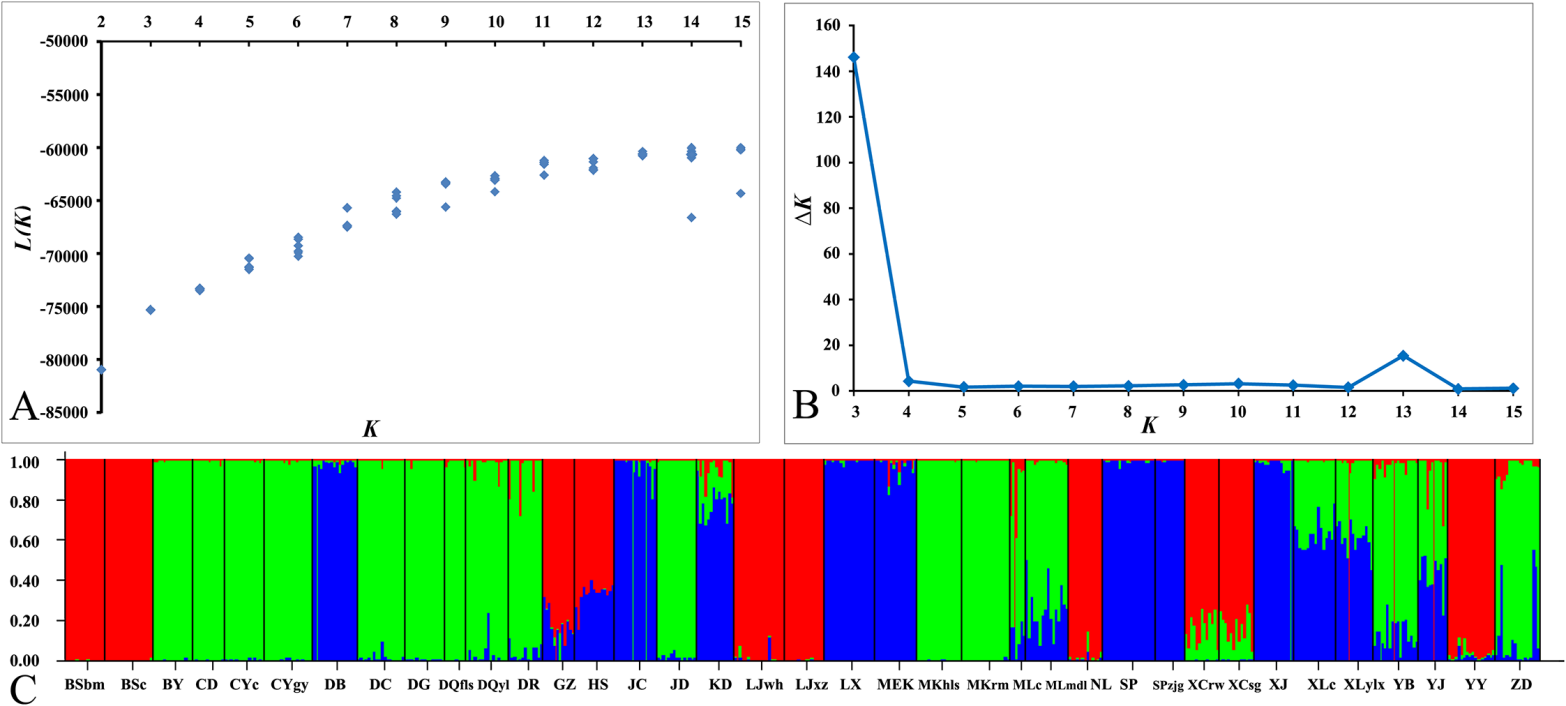
Genetic structure of *R*. *soulieana* based on Bayesian analysis. (A) Log probability of data L(k) as a function of k for 10 runs from k = 2 to 15. (B) Rate of change in the probability between successive runs, Δk, as a function of k. (C) Assignment of 556 individuals into 3 (k = 3) genetically distinguished groups. Each individual is represented by a vertical bar partitioned into 3 colored segments that denote the individual’s estimated membership fraction in 3 clusters.

Genetic structure analysis (Bayesian clustering) revealed that the highest estimation of the posterior log probability given the number of clusters chosen, L(k), occurred at k = 3 (Fig 6). Considering the rate of change between successive runs, the maximum value of ΔK was more clearly associated with k = 3. Thus, all of the individuals could be potentially assigned into 3 groups (Fig 2). The first group included almost all individuals of population BY, CD, CYc, CYgy, DC, DG, DQfls, DQyl, DR, JD, MKhls and MKrm, over 80.0% individuals in population YB and ZD, 70.0% individuals in population MLc and MLmdl. The second group included almost all individuals of population DB, JC, LX, MEK, SP, SPzjg and XJ, about 80.0% individuals in population KD, 60% individuals in population XLc and XLylx, 40.0% individuals in population HS and YJ, about 15.0% individuals in population GZ, MLc, MLmdl, YB and ZD. The third group included almost all individuals of population BSbm, BSc, LJwh, LJxz, NL and YY, over 80% individuals in population GZ, XCrw and XCsg, about 70.0% individuals in population HS, 10.0% individuals in population MLc, and the remained very few individuals of other populations. Thus, the result of Bayesian analysis (Fig 6) showed that each population contained all the identified AFLP genotypes more or less, and that these three genotypes concentrated much more significantly in the populations along the Yalongjiang River.

According to Figs 5 and 6, NJ tree and Bayesian analysis produced highly consistent grouping of 37 populations. Almost all individuals of populations from Minjiang, Daduhe and Yalongjiang grouped together. Nearly all individuals of populations from upper Jinshajiang, upper Mekong River and the eastern Himalayan region grouped together. Others, including almost all individuals of populations from the northernmost part of the species range, and those in the southernmost part, grouped together. Only in the populations of YB, ZD, YJ, MLmdl and MLc, some individuals were assigned by both methods to populations from the other geographic groups. The populations YB and MLc were grouped with populations from the most southern distribution in the NJ tree, but most individuals in these populations were assigned to the group of upper Jinshajiang and upper Mekong River in the Bayesian analysis. The population ZD belonged to the group of upper Jinshajiang and Mekong River, but over 10% of its individuals were assigned to the group of Minjiang, Daduhe and Yalongjiang. The populations YJ and MLmdl belonged to the group of Minjiang, Daduhe and Yalongjiang in the NJ tree, while more than half of their individuals were assigned to the group of upper Jinshajiang and Mekong River.

### Relationship between geographic distance and genetic distance

**Fig 7:**
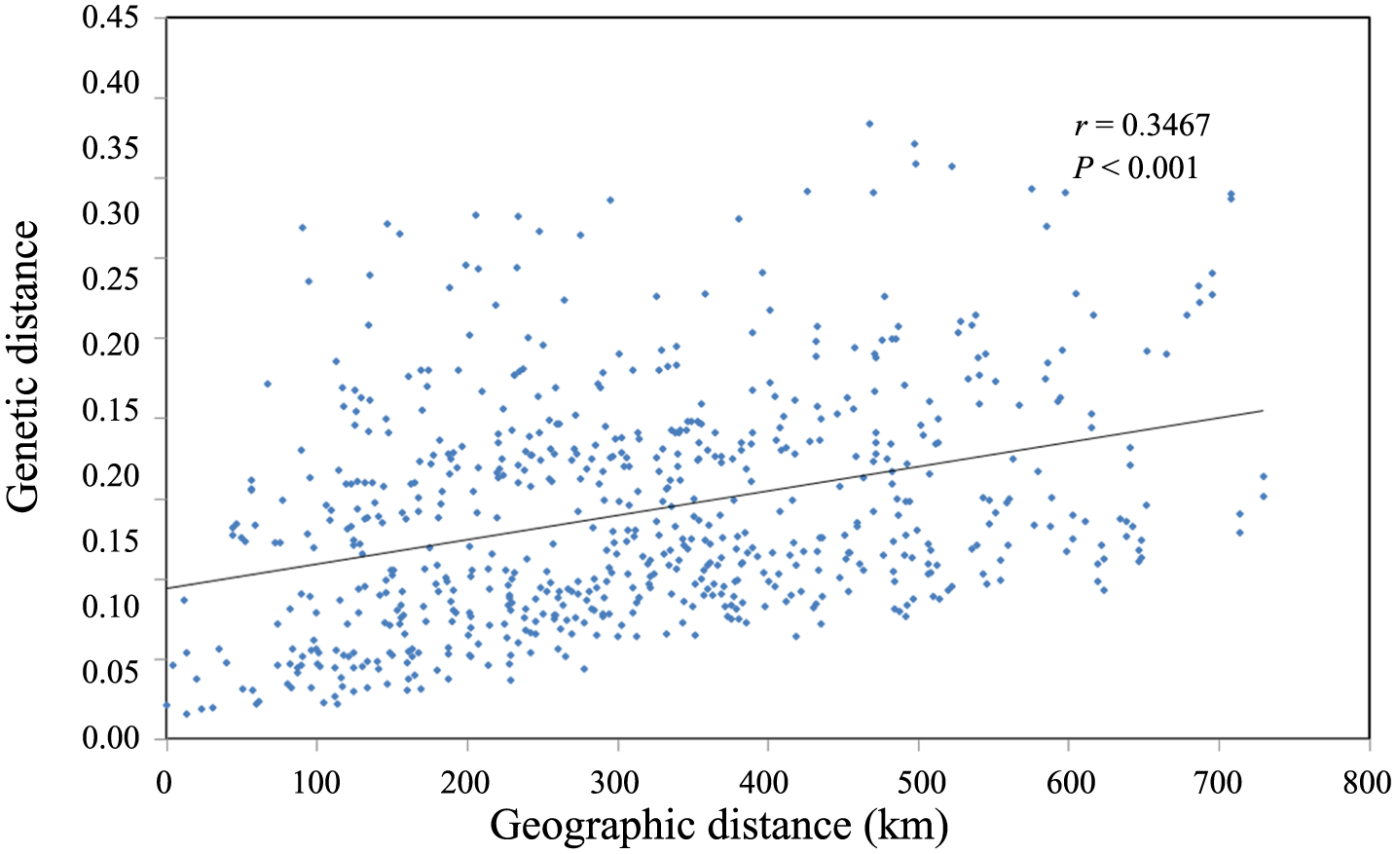
Significant and positive correlation between the Nei’s genetic distance and the geographic distance (km) of *R*. *soulieana*.

The Mantel test detected a positive and significant (r = 0.3467; P<0.001) correlation between Nei’s genetic diversity and geographic distance (km) in *R*. *soulieana* (Fig 7). This result implied that *R*. *soulieana* demonstrated a historical pattern of isolation-by-distance.

### Species genetic diversity and genetic structure (AMOVA)

The total heterozygosity (*H*t) of *R*. *soulieana* at the species level was 0.253±0.029; the heterozygosity value within populations (*H*s) at the species level was 0.139±0.011. The overall estimate of genetic structure, i.e., the gene differentiation coefficient (*G*_st_), is 0.451 (p<0.001). Genetic differentiation based on pairwise *F*_st_ comparisons between populations ranged from 0.016 (BSbm and BSc) to 0.384 (LJxz and BY). The gene flow (*N*m) per generation is 0.608. AMOVA revealed that 46.0% of the total variation resided among populations while 54.0% of the total variation resided within populations (Table 3), indicating that most of the genetic diversity occurred within populations.

**Table 3:** Analysis of molecular variance in *R*. *soulieana* with different groupings.

Dataset	Source of variation	*df*	Sum of squares	Variance components	Percentage of variation%
	Among populations	36	14340.31	24.6	46.0
Whole	Within populations	519	15078.93	29.1	54.0
	total	555	29419.24	53.7	
	Among groups	2	4668.98	11.0	19.2
Whole	Among populations within groups	34	9671.33	17.1	29.9
	Within populations	519	15078.93	29.1	50.9
	Total	555	29419.24	57.1	
	Among populations	11	2009.45	10.1	27.1
Group I	Within populations	173	4694.87	27.1	72.9
	total	184	6704.32	37.3	
	Among populations	11	2385.47	12.5	28.8
Group II	Within populations	168	5162.68	30.7	71.2
	total	179	7548.14	43.2	
	Among populations	12	5276.41	28.1	48.9
Group III	Within populations	178	5221.38	29.3	51.1
	Total	190	10497.79	57.4	

According to the result of the NJ phylogram, the populations of *R*. *soulieana* were clustered into 3 groups (Table 4). Total genetic diversity and genetic differentiation were higher in group III (*H* = 0.262, *I* = 0.404, *G*st = 0.464) than in group II (*H* = 0.206, *I* = 0.325, *G*st = 0.299) and in group I (*H* = 0.179, *I* = 0.284, *G*st = 0.281). When applied to the whole dataset, hierarchical AMOVA (Table 3) confirmed the significant differentiation among groups I, II and III (19.2% of the total variation), with the among-population and within-population variation components being with 29.9% and 50.9%, respectively. In group III, about half (48.9%) of the variation was among populations, while in groups II and I, among-population variation was less than 30.0% and most of the variation was within populations.

**Table 4:** Number of polymorphic bands (*NP*), the percentage of polymorphic loci (*P*), total heterozygosity (*H*_t_), Nei’s gene diversity (*He*) and Shannon’s information index (*I*), gene differentiation coefficient (*G*_st_) and gene flow (*N*_m_) in each group based on NJ tree.

Group	Populations	Individuals	*NP*	*P* %	*H*_t_ (St.Dev.)	*H*_e_(St.Dev.)	*I* (St.Dev.)	*G*_st_	*N*_m_
Group I	XLc, XLylx, YJ, MLmdl, KD, DB, XJ, JC, MEK, LX, SP, SPzjg	185	340	81.5	0.182 (0.031)	0.131 (0.017)	0.284 (0.246)	0.281	1.277
Group II	CYc, CYgy, DQyl, DQfls, DC, DR, JD, MKhls, CD, MKrm, DG, BY	180	355	85.1	0.207 (0.030)	0.145 (0.015)	0.325 (0.241)	0.299	1.171
Group III	BSc, BSbm, LJwh, LJxz, HS, GZ, XCsg, XCrw, YY, NL, YB, MLc, ZD	191	392	94.0	0.262 (0.172)	0.140 (0.012)	0.404 (0.227)	0.464	0.578
Total		556	408	97.8	0.253 (0.029)	0.145 (0.170)	0.339 (0.011)	0.451	0.608

## Discussion

### Moderately high level of genetic diversity and high genetic differentiation

The genetic diversity based on AFLPs of *R*. *soulieana* was moderately high at the species level, despite that it was also relatively lower at the population level. The total heterozygosity (*H*t) was 0.253, which was comparable to other outbreeding woody perennials having a moderate to high level of AFLP genetic diversity, e.g., *Rosa arvensis* (0.237) [39] of the same genus, *Junipers oxycedrus* subsp. *macrocarpa* (0.2097) [40] from the Iberian Peninsula, *Cedrela balansae* (0.222) [41] from Northwestern Argentina, and *Buddleja crispa* (0.3135) [42] from the Himalaya-Hengduan Mountains region. The high levels of diversity at the species level can be attributed to a number of factors including geographic distance, breeding system, gene flow etc. [18, 43], besides AFLP itself surveyed numerous DNA loci across the entire nuclear genome by combining two parents. First, the distribution of *R*. *soulieana* covers almost the whole Hengduan Mountains range, although it is endemic to this area [44]. The large geographic distances among populations might be one reason for its moderately high level of genetic diversity [45]. Second, *R*. *soulieana* is an entomophilous plant pollinated by bees, *Bombus* and butterflies. Although both geitonogamy selfing and xenogamy exist in this species, xenogamy can get much more achenes in each hip [46], indicating that outbreeding is its main way of reproduction. Outcrossing species commonly have higher levels of genetic diversity than selfing congeners [18]. Third, *R*. *soulieana* is a long-lived woody perennial, and long-lived woody perennials in general have higher genetic diversity at the population level than short-lived perennials and annuals [18, 47–48].

As far as genetic structure is concerned, more than half (54.0%) of the genetic diversity of *R*. *soulieana* was within the populations. The gene differentiation coefficient (*G*_st_) was 0.451, which was much higher than that of most outcrossing species (approximately 0.20–0.23) and lower than that of inbreeding species (0.50–0.59) analyzed with dominant markers [18, 49]. The high level of genetic differentiation among populations was consistent with its life form, breeding system and reproductive characteristics [46], especially the way of seed dispersal. Most seeds of *R*. *soulieana* are dispersed close to the mother plant by gravity [13], which will cause reduced seed flow. Besides, birds may play a role in the dispersal of seeds of *R*. *soulieana*, because hips of other wild roses are known to be widely dispersed by birds in Europe (*R*. *canina* [50] and *R*. *arvensis* [39]), North America (*R*. *multiflora* [51]), and also in the Tibetan area (*R*. *sericea* [52]). This might have also contributed to its high level of genetic differentiation among populations, as proved in *R*. *multiflora* [51]. In addition to the breeding system, other factors contributing to high genetic differentiation in *R*. *soulieana* can be isolation by geographic barriers. The complex topography and strong environmental heterogeneity in the East Himalaya-Hengduan Mountains region, especially the series of parallel mountain ranges dissected by deep river valleys that run from north to south, can act as physical barriers to gene flow [53]. The *N*m of *R*. *soulieana* (0.608) was less than unity, indicating that there was restricted gene flow at the species level. Furthermore, the positive and significant correlation between genetic and geographic distances further explained the relatively high level of genetic differentiation among populations due to its wide distribution in the Hengduan Mountains.

### Inconsistency between morphological variation and AFLP genotyping

Morphological characteristics have traditionally been used in taxonomy and their application has often led to conclusions later confirmed by molecular markers. Nevertheless, morphological characteristics are not always able to detect taxonomic diversity [54]. Although 4 intraspecific varieties have been distinguished according to variable morphological characteristics, this intraspecific classification was not confirmed by detected cpDNA haplotype variation [16]. Based on the Bayesian analysis and NJ clustering, the genotypes of *R*. *soulieana* were clustered into 3 groups. The first group included several populations distinguished as *R*. *soulieana* var. *soulieana* and most populations of *R*. *soulieana* var. *yunnanensis*. The second group included most populations of *R*. *soulieana* var. *soulieana* and the only population of *R*. *soulieana* var. *sungpanensis*. The third group included the only population of *R*. *soulieana* var. *microphylla* (BSbm), several populations of *R*. *soulieana* var. *soulieana* (HS, GZ, XCrw and XCsg) and some populations of *R*. *soulieana* var. *yunnanensis*, even though their relationship was not so close to each other, as shown in the results of PCoA (Figs 3 and 4). These results suggest that the morphology-based intraspecific taxonomy is not supported at the nuclear DNA level and the intraspecific varieties are not evolutionary significant units sensus Avise (2000) [19].

Intraspecific morphological variation in plants can arise from many factors, including cytological variation within the specie and hybridization with other co-occurring congeners. However, *R*. *soulieana* is a diploid with 14 chromosomes and no variation has been reported within the species [55, 56]. As for hybridization, population SP, the only population of *R*. *soulieana* var. *sungpanensis*, with much larger leaflets and more florets, over-laps in distribution with *R*. *filipes* Rehd. et Wils. from the same section. They might hybridize, but population SP was grouped in our study with populations geologically far away from the *R*. *filipes* range. Thus, the morphological diversification in *R*. *soulieana* is neither caused by cytological variation nor by hybridization. This diversification could result from plastic responses to the variation in topography and climate in the Hengduan Mountains, as roses are notorious for their phenotypic plasticity, e.g., *R*. *arvensis* [39], *R*. *damascena* [57], *R*. *canina* complex [58] and *R*. *villosa* complex [59]. Besides, heritable phenotypic variation in natural populations can be due to stable epigenetic variation and can also play a role in plant adaptation and evolution, as in *Limonium* species with different ploidy levels [60] and in allotetraploid sibling orchids [61]. Whether or not the morphological variation observed in *R*. *soulieana* is caused by epigenetic modifications of DNA or histones, needs further studying.

### Possible dispersal and divergence implications

According to the spatial genetic structure of *R*. *soulieana*, it can be seen that the Yalongjiang River played a very important role in the dispersal and divergence of this wild rose species. The 3 AFLP genotypes significantly concentrated in the populations along the Yalongjiang River.

Jian et al. [16] postulated that northwestern Yunnan and Yalongjiang valley were the refuge and centers of adaptive divergence for *R*. *soulieana* during the Quaternary climate oscillations based on cpDNA phylogeography. This was confirmed in this study and it could also be proposed that the ancient *R*. *soulieana* of the third group moved upnorth along the Yalongjiang River, and then dispersed east to Heishui (HS) and west to Baxoi (BS) (Fig 2). This is suggested by the following lines of evidence. First, genetic diversity of most populations near northwestern Yunnan and the lower part of the Yalongjiang River, e.g., JD, ZD, DR, LJwh, CYc, MLmdl and YJ, and those belonging to the third group, such as NL, XCrw, XCsg and YY, was generally higher than that of other northern populations belonging to the third group, such as BSbm, BSc, GZ and HS, because of founder effects due to dispersal or expansion during the Quaternary, usually observed in comparisons of refuge areas with other newly colonized locations [62], as in *Pedicularis longiflora* [63] and *Arcterica nana* [64]. Second, the seeds of *R*. *soulieana* can be dispersed by birds as stated above. It is much easier for the birds with long distance migration to disperse the seeds of *R*. *soulieana* in the much plainer northern part of the Hengduan Mountains [65, 66] than in the middle part.

Climatically, the Hengduan Mountains Region can be divided into Qinghai-Xizang Plateau Cold Climate and Southeastern Monsoon Climatic Realms [66]. The Mountain Warm Zone in the Southeastern Monsoon Climatic Realm acts as a link between the two climatic realms [66]. The first part of distribution of *R*. *soulieana* in the east of the Yalongjiang River belongs to the Mountain Warm Zones in the Southeastern Monsoon Climatic Realm, while the second part in the west of Yalongjiang River belongs to the Qinghai-Xizang Plateau Cold Climate Realm (Fig 2). Interestingly, the first and second genetic groups of *R*. *soulieana* revealed by the NJ tree are located geographically at the eastern and western sides of the Yalongjiang River, respectively. Thus, the river could have acted as a vicariance line for the species division. Gene flow among populations within each group was higher than the gene flow among all populations (*N*m of the east = 1.277, *N*m of the west = 1.171, *N*m of the species = 0.608). The populations between the first group (Group I) and the second group (Group II) had a very limited gene flow. According to the result of Bayesian analysis, genotypes of both groups concentrated significantly in the populations along the Yalongjiang River belonging to the third group (Group III)(Figs 2 and 6C), and only a very small percentage of individuals of one group crossed the river and settled in the populations of the other group. Because ecological characteristics such as temperature, amount and frequency of rain and other climatic conditions may have an effect on inducing inter- and intraspecific variation [67], the apparent variation may be due to the environment in which these plants grow, as observed in *Rosa persica* [68]. Thus, the two groups of *R*. *soulieana* might be the result of allopatric divergence due to long period of adaptation to different climatic conditions at either bank of the Yalongjiang River.

### The implication for the utilization and preservation of *R*. *soulieana*

According to the genetic structure of *R*. *soulieana* based on AFLP genotypes in this study and the cpDNA haplotypes reported by Jian et al. [16], it is preferable to collect and preserve the populations in southeastern Tibet, northwestern Yunnan and along the Yalongjiang River during wild rose germplasm collection. Furthermore, when choosing this wild rose as a plant for the ecosystem restoration of the dry and hot valleys in the Hengduan Mountains, it is better to choose propagation materials from the east of the Yalongjiang River for the revegetation of areas in the Mountain Warm Zones, and to choose propagation materials from the west of Yalongjiang River for the revegetation of areas in the Qinghai-Xizang Plateau Cold Climate Realm.

## Supporting Information

S1 Table Allele report for EcoRI- AAG / MseI- CAT. (XLSX)

S2 Table Allele report for EcoRI- AAG / MseI- CGT. (XLSX)

S3 Table Allele report for EcoRI- AAG / MseI- CTC. (XLSX)

S4 Table Allele report for EcoRI- AAG / MseI- CTT. (XLSX)

S5 Table Pairwise genetic distances between populations (*F*_st_) among 37 populations. (XLSX)
